# Insidious Primary Mediastinal Large B-Cell Lymphoma in a Young Female: A Case Report and Literature Review

**DOI:** 10.1155/crom/9983831

**Published:** 2025-04-26

**Authors:** Neil Gambhir, Paul Youn, Moein Bayat Mokhtari, Patricia Lin Kwan, Thida Aye, Wan Ling Lam

**Affiliations:** Department of Medicine, Lenox Hill Hospital, New York, New York, USA

**Keywords:** cardiac tamponade, palpitations, primary mediastinal large B cell lymphoma

## Abstract

Primary mediastinal large B-cell lymphoma (PMBCL) is a highly aggressive malignancy primarily observed in female patients during their third decade of life. This rare condition, with an incidence of 0.4 per million, traditionally presents with B symptoms or compressive-based sequela such as SVC syndrome or respiratory distress. In this report, we present the case of a young female who presented for palpitations and tachycardia without cardiopulmonary compressive–based symptoms diagnosed with a large 16-cm infiltrative PMBCL localized in the left ventricle.

## 1. Introduction

Primary mediastinal large B-cell lymphoma (PMBCL) is a mature B-cell lymphoma of a thymic origin predominantly encountered in young female patients in the third decade of life. PMBCL represents 7% of all diffuse large B-cell lymphomas, often manifesting as a large anterior mediastinal mass that compresses adjacent structures. This frequently leads to a rapid progression of compression-related symptoms including dyspnea, cough, airway compromise, superior vena cava (SVC) syndrome, and in severe cases, compression of the great vessels [1]. In fact, a recent review reported an incidence as high as 57% for patients with PMBCL presenting with SVC syndrome [2, 3]. Moreover, since PMBCL is a variant of Hodgkin's, patients may report B-symptoms, such as night sweats and weight loss.

We present the case of a 25-year-old female who presented with palpitations initially attributed to anxiety but was later diagnosed with a large invasive pericardial PMBCL involving the left ventricle. She received treatment with etoposide, prednisone, vincristine, cyclophosphamide, and doxorubicin (E-POCH). This case is noteworthy due to the rare presentation of PMBCL with cardiac involvement, and we aim to provide valuable insights into the diagnostic challenges, clinical course, and management of this condition, contributing to a better understanding of its clinical manifestations and improving awareness of its potential cardiac complications.

## 2. Case

**Figure 1:**
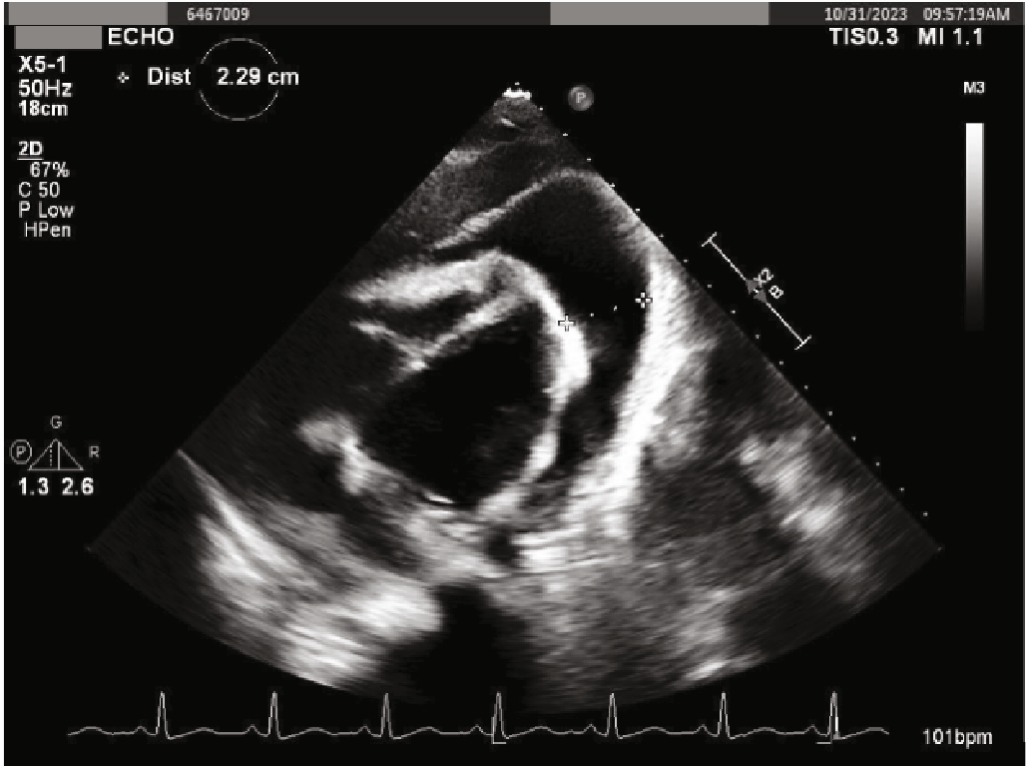
Outpatient echocardiogram demonstrating a 16-cm mass.

The index patient is a 25-year-old female with a past medical history significant for gastroesophageal reflux who initially presented to an outside medical facility with complaints of palpitations and tachycardia. The patient was monitored and found to have sinus tachycardia. Workup at the outside medical facility was unremarkable. She was diagnosed with anxiety-mediated symptoms, prescribed lorazepam, and advised to follow up with her primary care physician. Following discharge, the patient experienced 2 weeks' worth of progressively worsening dyspnea, initially on exertion and later at rest, accompanied with a 5-lb weight loss, dry cough, palpitations, and persistent tachycardia as noted on her Apple Watch (115–120 bpms). Following the exacerbation of these symptoms, she was referred to a cardiologist, where a transthoracic echocardiogram revealed a substantial pericardial effusion suggestive of tamponade, along with the visualization of a vascularized mass at the heart's angle. Subsequently, she was promptly admitted to our facility (Figure 1).

**Figure 2:**
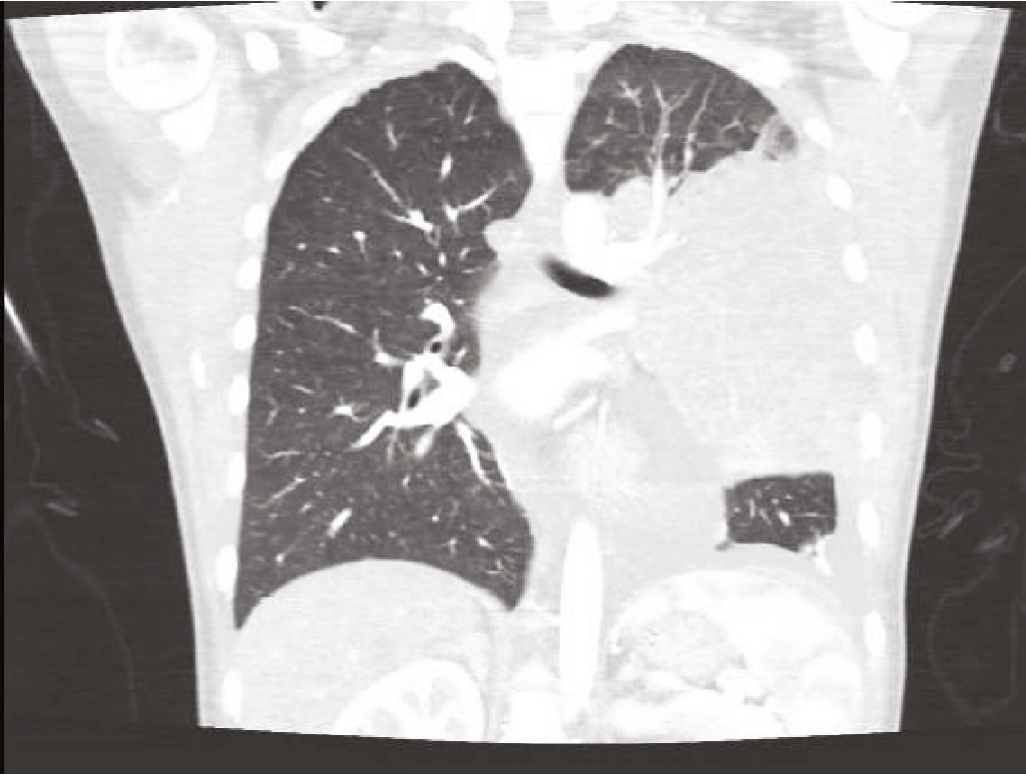
CT scan demonstrating a mediastinal mass narrowing the left mainstem bronchus and involving the pericardium and myocardium of the left ventricle.

Upon initial evaluation, the patients' vitals were significant for tachycardia (110 bpm) and a chest CT was obtained demonstrating a large mediastinal mass (16 cm) narrowing the left mainstem bronchus and distal airway that involved the pericardium and myocardium of the left ventricle (Figure 2). At the time, there were no discernible physical signs indicative of cardiac tamponade, and the rest of the physical examination was normal. Preliminary lab work was significant for mild anemia (Hgb 11.0 g/dL) and thrombocytosis (Plt 488 10^3^/*μ*L).

Given her presentation, primary mediastinal lymphoma was the leading consideration, though thymic neoplasm, germ cell tumor, and less likely, metastatic disease were also considered. Infectious and granulomatous etiologies such as tuberculosis, histoplasmosis, or sarcoidosis were thought to be less probable given the rapid progression and lack of an immunocompromised state.

Given these findings, a multidisciplinary approach was taken involving our structural heart, internal medicine, oncology, thoracic surgery, and interventional radiology teams. Image-guided biopsy and pericardiocentesis with drain placement were performed. The biopsy revealed lymphocytes and inflammatory cells on touch prep and the pericardiocentesis indicated lymphocyte-predominant exudative pericardial effusion (997 nucleated cell, 66% lymph, LDH ratio 1.42 (696/488), and TP ratio 0.65 (4/7/7.2).

Following drainage, a repeat transthoracic echocardiogram showed a significant improvement of the effusion and clear vascularization of an extracardiac mass noted anterior to the heart. Preliminary cytology studies and PET-CT further supported the likely diagnosis, indicating a high-grade lymphoma. Immunohistochemistry was performed, revealing that the tumor cells were negative for desmin, myogenin, S100, SOX10, ERG, MDM2, CD117, and ALK (D5F3). As such, urgent initiation of chemotherapy with E-POCH was given due to the presence and position of the bulky mass in a critical anatomical location. The patient was transferred to medical ICU during the chemotherapy initiation phase for closer monitoring and comprehensive care and to mitigate the potential risks of tumor lysis syndrome, vascular perforation, and arrythmia. Afterwards, the patient tolerated the treatment well and was safely discharged.

## 3. Follow-Up

**Figure 3:**
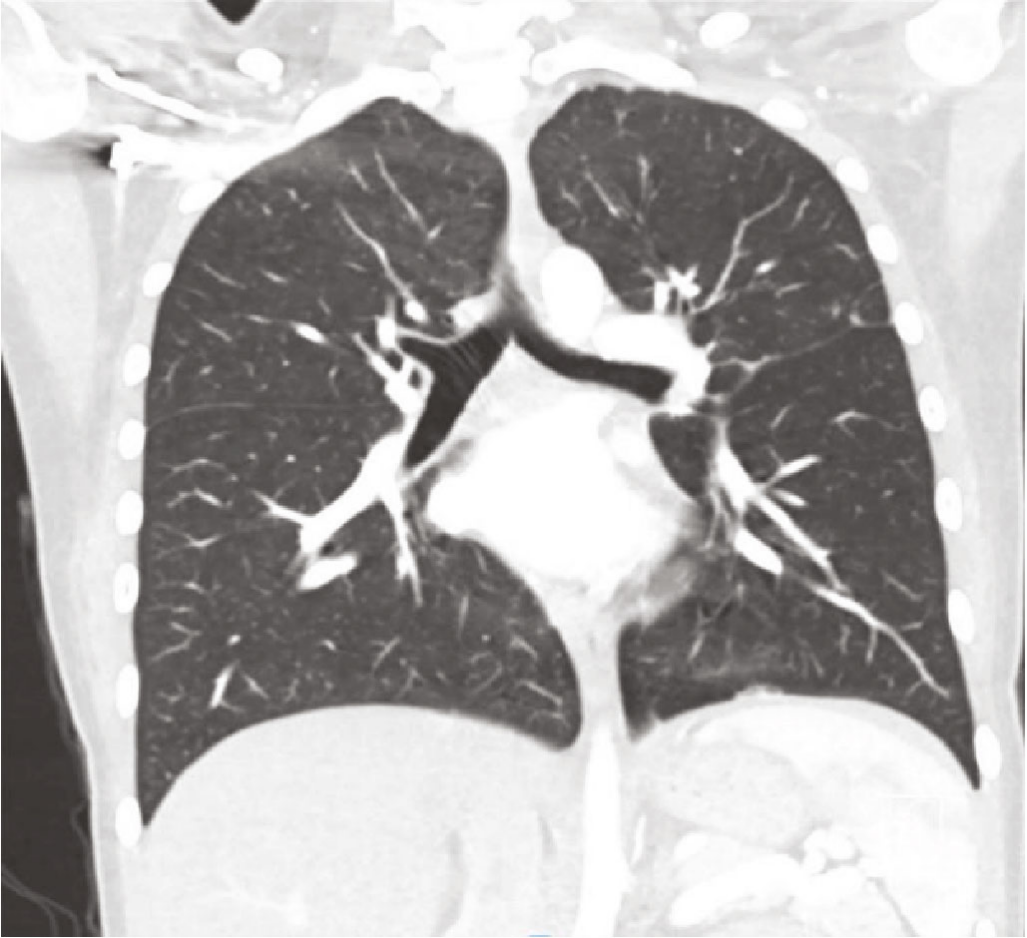
CT scan at the latest follow-up demonstrating a significant reduction in disease burden.

At the last follow-up, the patient has completed three out of six rounds of chemotherapy with resolution of her cardiopulmonary symptoms. Significant decreases in tumor burden have also been appreciated on repeat CT imaging (Figure 3).

## 4. Discussion

First described by Van-Dam in the early 1970s, PMBCL is a rare and aggressive subtype of diffuse large B-cell lymphoma (DLBCL), comprising approximately 2%–4% of all non-Hodgkin lymphomas. It primarily affects young adults, with a slight female predominance, and originates from thymic B-cells within the anterior mediastinum [3]. Patients with PMBCL commonly present with symptoms related to local mass effect, including dyspnea, cough, dysphagia, and, in severe cases, airway compression or SVC syndrome due to vascular obstruction.

However, our patient's presentation was atypical, as she initially exhibited persistent tachycardia and palpitations, which were initially attributed to anxiety. Only later did she develop progressive dyspnea and weight loss, without overt signs of cardiopulmonary compression. Imaging ultimately revealed a large mediastinal mass with direct infiltration into the pericardium and myocardium, an unusual finding that posed unique diagnostic and therapeutic challenges. Unlike the more common presentations involving SVC obstruction or tracheobronchial compression, her case underscores the need for clinicians to maintain a high index of suspicion for PMBCL even in the absence of classic signs.

The diagnosis of PMBCL requires a comprehensive approach, beginning with a thorough history and physical examination, though exam findings are often nonspecific. Imaging plays a pivotal role, with CT and PET-CT revealing a bulky, hypermetabolic anterior mediastinal mass. Laboratory evaluation may show elevated inflammatory markers, anemia, or thrombocytosis, but definitive diagnosis relies on biopsy with histopathological and immunohistochemical analysis. Our patient's biopsy confirmed a high-grade lymphoma, supported by immunohistochemical findings showing tumor cells negative for desmin, myogenin, S100, SOX10, ERG, MDM2, CD117, and ALK D5F3, ruling out other mediastinal neoplasms.

The standard treatment for PMBCL is dose-adjusted E-POCH, which has shown superior efficacy compared to traditional CHOP regimens. Given our patient's cardiac involvement, treatment posed additional challenges due to the potential risk of chemotherapy-induced arrhythmias and cardiac complications. Careful multidisciplinary coordination was essential in balancing the urgency of treatment initiation with the risks associated with myocardial infiltration.

Similar to our patient's presentation, Rogowitz et al. [4] described a case of a 23-year-old female who presented with SVC syndrome. She was diagnosed with infiltrative PMBCL through the right atrium and tricuspid valve, resulting in regurgitation. The patient responded positively to chemotherapy on R-CHOP. On the more invasive side of the spectrum, Carro et al. [5] reported a case of a 19-year-old primigravid female who presented with cardiogenic shock and SVC syndrome attributed to a large mass. Unfortunately, the patient's malignancy was unresponsive to chemotherapy, requiring an extensive hospital course involving debulking surgery and ECMO.

While the primary demographic affected by PMBCL is that of young females, cases involving males have been documented in recent years. Jomma et al. reported a case of a 25-year-old man who experienced sudden-onset dyspnea followed by loss of consciousness and subsequent sudden death [6]. The autopsy revealed a PMBCL mass (> 10 cm) compressing his mediastinal organs and trachea, which led to his acute respiratory failure. Of particular interest is the work of Wang et al. [7] who described the case of a 43-year-old Chinese male with respiratory symptoms. His malignancy was initially misdiagnosed as tuberculosis following an ultrasound exam, further highlighting the complexity of diagnosing PMBCL.

## 5. Conclusion

PMBCL is a rare but treatable malignancy when diagnosed early. While it typically presents with cardiopulmonary symptoms, this case highlights the importance of recognizing atypical presentations, such as palpitations and persistent tachycardia, which initially led to a misattributed diagnosis. The identification of pericardial effusion and myocardial involvement further distinguishes this case, emphasizing the need for a high index of suspicion and thorough evaluation. This case contributes to the literature by underscoring the diverse clinical spectrum of PMBCL and the critical role of imaging and histopathology in guiding timely diagnosis and treatment.
